# Genome-wide mutation analysis of *Helicobacter pylori* after inoculation to Mongolian gerbils

**DOI:** 10.1186/s13099-019-0326-5

**Published:** 2019-09-21

**Authors:** Rumiko Suzuki, Kazuhito Satou, Akino Shiroma, Makiko Shimoji, Kuniko Teruya, Takashi Matsumoto, Junko Akada, Takashi Hirano, Yoshio Yamaoka

**Affiliations:** 1 Department of Environmental and Preventive Medicine, Oita University Faculty of Medicine, 1-1 Idaigaoka, Hasama-machi, Yufu, Oita 879-5593 Japan; 2 Okinawa Institute of Advanced Sciences, 5-1 Suzaki, Uruma, Okinawa 904-2234 Japan; 3 Department of Medicine-Gastroenterology, Baylor College of Medicine, 2002 Holcombe Blvd., Houston, TX 77030 USA; 4 Global Oita Medical Advanced Research Center for Health, 1-1 Idaigaoka, Hasama-machi, Yufu, Oita 879-5593 Japan

**Keywords:** *Helicobacter pylori*, Mongolian gerbil, Animal model, Genome comparison, Adaptive mutation, Protein structure

## Abstract

**Background:**

*Helicobacter pylori* is a pathogenic bacterium that causes various gastrointestinal diseases in the human stomach. *H. pylori* is well adapted to the human stomach but does not easily infect other animals. As a model animal, Mongolian gerbils are often used, however, the genome of the inoculated *H. pylori* may accumulate mutations to adapt to the new host. To investigate mutations occurring in *H. pylori* after infection in Mongolian gerbils, we compared the whole genome sequence of TN2 wild type strain (TN2wt) and next generation sequencing data of retrieved strains from the animals after different lengths of infection.

**Results:**

We identified mutations in 21 loci of 17 genes of the post-inoculation strains. Of the 17 genes, five were outer membrane proteins that potentially influence on the colonization and inflammation. Missense and nonsense mutations were observed in 15 and 6 loci, respectively. Multiple mutations were observed in three genes. Mutated genes included *babA*, *tlpB*, and *gltS*, which are known to be associated with adaptation to murine. Other mutations were involved with chemoreceptor, pH regulator, and outer membrane proteins, which also have potential to influence on the adaptation to the new host.

**Conclusions:**

We confirmed mutations in genes previously reported to be associated with adaptation to Mongolian gerbils. We also listed up genes that mutated during the infection to the gerbils, though it needs experiments to prove the influence on adaptation.

## Background

*Helicobacter pylori* (*H. pylori*) is known to a risk factor of various gastrointestinal diseases [1–4]. Previous studies investigated genetic diversification of *H. pylori* in the time course of chronic infection or transmission and revealed that the mutation rate of this bacterium is high [5–8].

However, *H. pylori* is well adapted to the human stomach but does not easily infect other animals. In search of a good animal model, experimental infection was attempted in Rhesus monkeys [9], mice [10], and Mongolian gerbils [11–16]. Genetic diversification of *H. pylori* in the infected animals was also studied [9, 10, 16–19].

Model animals are expected to respond to the stimulation in the similar manner to humans and be maintained on reasonable cost and handling efforts. Small rodent Mongolian gerbils develop similar symptoms to human by *H. pylori* infection as gastric inflammation, ulceration and cancer [13, 15, 20, 21]. Thus, they work as the good animal model.

We also used Mongolian gerbils as the model animal and discovered that *babA* expression in *H. pylori* initially increased upon infection but reduced over time, then lost after 6 months [22] and that infection with *oipA* or *babA* mutants resulted in significantly reduced cytokine levels but *alpAB* mutant did not infect Mongolian gerbils [22].

Earlier studies used PCR to investigate changes in genes during animal infection. However, DNA sequencing advancements enabled the extensive exploration of mutations by sequencing bacterial genomes before and after infection [16, 19]. Here, we used the whole genome sequence of TN2 wild type (TN2wt) as a reference and sequenced short reads from three derivative strains to identify genomic mutations during infection in Mongolian gerbils. We detected mutations in agreement with previous studies and identified new mutations that may be associated with adaptation of the bacteria to different hosts.

## Methods

### Inoculation, euthanasia and isolation of *H. pylori*

We inoculated TN2 wild type strain [21] (TNwt) to Mongolian gerbils as described in our previous paper [12]. Six-week-old male Mongolian gerbils (MGS/Sea; Harlan Sprague Dawley) were orogastrically inoculated 3 times (days 0, 1, 2) with 1.0 mL of *H. pylori* (10^9^ colon-forming units/mL) or sterile brain–heart infusion (BHI) broth using gastric intubation needles after 16 h of fasting [10]. No specific pretreatments were administered prior to orogastric *H. pylori* inoculation. Inoculated Mongolian gerbils were sacrificed after 1 month (TN2-1M), 3 months (TN2-3M), and 6 months (TN2-6M). At necropsy, an ~ 1-mm^2^ piece of gastric mucosa from the antrum was collected for culturing of *H. pylori* and subsequent DNA extraction.

### Bacterial culture and DNA extraction

*Helicobacter pylori* were cultured on confluent plates expanded from a single colony under microaerobic conditions (12% CO_2_) at 37 °C. Bacterial DNA was extracted from the plates using a commercially available kit (QIAGEN Inc., Valencia, CA, USA).

### Sequencing of the genomic DNA

The whole genome sequence of TN2wt was provided by our collaborator at the Okinawa Institute of Advanced Sciences. The whole-genome sequencing of TN2wt was carried out using the PacBio RS II (Pacific Biosciences, Menlo Park, CA) platform. De-novo assembly was performed using the hierarchical genome assembly process (HGAP) workflow [23], including consensus polishing with Quiver v. 2.3.3. By this workflow, the complete genome sequence of TN2wt was obtained. Annotation was performed by MiGap service provided by National Institute of Genetics. The genome DNA of *H. pylori* strains retrieved from the Mongolian gerbils were sequenced by HiSeq2000 (paired end, 2 × 100 bp). DNA was quantified by Qubit fluorometric method (Thermo Fisher Scientific). DNA purity was assessed by the UV absorbance ratio at 260/280 with 1.8–2.0. Finally, 500 ng of DNA input was used for DNA library preparation. The numbers of reads obtained were 13,574,248, 14,583,596, and 13,938,018 for TN2-1M, TN2-3M, and TN2-6M, respectively; 99.69%, 99.74%, and 99.75% of the reads mapped to the reference TN2wt genome, resulting in average mapping depths of 758.8, 815.7, and 779.6 for TN2-1M, TN2-3M, and TN2-6M, respectively. The coverage of the reference genome was 100% in the all strains.

### Data analysis

Short read data of genomic DNA from the retrieved strains (TN2-1M, TN2-3M, and TN2-6M) were mapped to the complete genome sequence of TN2wt using Genomics Workbench v. 7.0.4 (CLC QIAGEN) with default parameter setting. We also attempted de-novo assembly, but the assembly produced around 30 contigs and the total length was shorter than the original genome. Therefore, we used the reference mapping results for the analysis. We selected non-synonymous mutations that were identified in more than 90% of the mapped reads. If available, protein structure data were downloaded from PDB (https://www.rcsb.org/) [24, 25] and the location of the mutated locus was visualized by Chimera v. 1.10.2 [26].

## Results and discussion

### Non-synonymous mutations in the retrieved strains

**Fig. 1:**
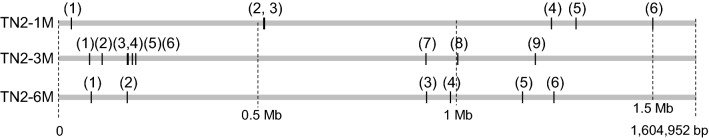
Schematic figure of mutation loci in each strain. Numbers in the parentheses correspond with those in Table 1

Compared with the original TN2wt genome, strains TN2-1M, TN2-3M, and TN2-6M had 6, 9, and 6 non-synonymous mutations, respectively (Table 1, Fig. 1). These mutations were resided in 17 genes. In accordance with our previous report [10], 5 of the 17 genes were outer membrane proteins that potentially influence on colonization and inflammation.

**Table 1:** Mutations observed in outcome strains

Strains		Position	Mutation	Depth	Ratio	Gene	Amino acid change
TN2-1M	(1)	31255	C → A	1025	99.7	Outer membrane protein (*hefG*)	A61S
	(2)	517741	G → A	569	99.7	Glutathione-regulated potassium-efflux system protein (*kefB*)	N232S
	(3)	517792	T → C	676	99.7		A249V
	(4)	1241193	Insertion	625	96.6	Outer membrane protein (*hofH*)	Frameshift without stop
	(5)	1297623	A → G	776	99.5	Urease accessory protein (*ureI*)	H131R
	(6)	1496148	A → C	529	95.1	Glutamate permease (*gltS*)	W131G
TN2-3M	(2)	112286	G → A	866	93.6	Dinucleoside polyphosphate hydrolase	R139C
	(5)	188008	C → G	766	100.0	Type II restriction enzyme R protein (*hsdR*)	R173T
	(6)	194568	G → T	774	99.6	Uncharacterized protein	G201W
	(7)	926807	Insertion	773	97.0	cag pathogenicity island protein (*cag8*)	Stop at 136th codon
	(8)	1007324	A → G	867	99.1	Outer membrane protein (*hopB*)	T123A
	(9)	1202841	G → A	780	99.9	F0F1 ATP synthase subunit alpha	P470L
TN2-6M	(3)	935451	C → T	691	99.9	P-type DNA transfer ATPase (*virB11*)	H314Y
	(4)	989679	Deletion	629	95.0	Outer membrane protein (*babA*)	Stop at 93th codon
	(5)	1174908	C → A	570	94.4	Lipopolysaccharide biosynthesis proteins	G154W
	(6)	1251850	Insertion	605	91.8	Outer membrane protein	Stop at 305th codon
TN2-3M	(1)	87451	Deletion	853	95.2	Oligopeptide ABC transporter periplasmic oligopeptide-binding protein (*oppA*)	Stop at 464th codon
TN2-6M	(1)			791	96.3		
TN2-3M	(3)	175008	G → T	591	97.8	Methyl-accepting chemotaxis protein (*tlpB*)	G26W
	(4)	175755	G → T	795	99.9		G275W
TN2-6M	(2)	175691	Deletion	810	99.9		Stop at 256th codon

Position indicates the location of the mutation in the TN2 genome. Depth and ratio represent number of reads that covered the locus and percentage of the mutated reads, respectively. Numbers in the parentheses correspond with those in Fig. 1

Some genes had multiple mutations. TN2-1M had two missense mutations in *kefB* and single missense mutation in other three genes. A nucleotide insertion in *hofH* of TN2-1M (1290th nucleotide in the gene) caused frameshift, however, it did not cause a premature stop codon. Instead, the frameshift delayed the occurrence of a stop codon and elongated the gene 15 bp. Consequently, mutations observed in TN2-1M were all missense. *KefB* is a component of potassium ion (K^+^) transportation system that regulates cytoplasmic pH and influence on bacterial growth and survival [27]. *UreI* is a pH-gated urea channel that enable *H. pylori* to colonize in acidic environment [28–30]. Missense mutations in these genes might change reactivity to pH fluctuation. *GltS* is a Glu-specific transporter and known also to be essential for colonization of *H. pylori* in Mongolian gerbils [31, 32].

TN2-3M contained seven missense and two nonsense mutations. Nucleotide deletion in *oppA* that leads to the premature stop codon was observed both in TN2-3M and TN2-6M. *OppA* is one of the ABC-type transporter genes for oligopeptide transport. Previous in-vitro study reported that disruption of *oppA* did not significantly change the growth of the mutant from the wild type [33]. This may suggest that the nonsense mutation in *oppA* was allowed because this gene is not essential for growth. Another possibility is that loss of *oppA* is neutral in vitro or in the originated human stomach but rather advantageous in the Mongolian gerbil stomach. Considering that the nonsense mutation of *oppA* was observed both in TN2-3M and TN2-6M, the latter hypothesis is also probable.

TN2-6M contained two missense and four nonsense mutations. In this strain, *babA*, *oppA*, *tlpB*, and outer membrane protein had nonsense mutations. As for *tlpB*, two missense mutations were also observed in TN2-3M. *TlpB* and *babA* are known to be involved with *H. pylori* adaptation to Mongolian gerbils. Our previous study revealed that infection with mutated *babA* reduced cytokine levels and inflammatory cell infiltrations of the host [22] and that *babA* expression disappeared 6 months after inoculation to Mongolian gerbils [12]. *TlpB* is a chemoreceptor that detect acidity and urea [34, 35]. Similar to *babA*, mutants lacking *tlpB* colonized as good as wild type but caused less inflammations in the stomach of mice and Mongolian gerbils [36, 37]. *TlpB* accepts posttranslational regulation by small RNA that targets guanin repeat (G-repeat) upstream of the gene [38]. Because expression of *tlpB* is affected by the G-repeat length, we counted the G-repeat length of our strain. The lengths were 12 for TN2wt, TN2-1M, and TN2-6M and 11 for TN2-3M, which are associated with low level of *tlpB* expression [38].

Mutations in *oppA* and *tlpB* have also been reported [19] (Table 2), but the inoculated animal in this study was a mouse. There were no genes in common with another genome study using the Mongolian gerbil as a model [16]. Another research group compared the *H. pylori* genome before (PMSS1) and after (SS1) inoculation [19]. They reported that *oppA* was disrupted in the original strain; we also observed disruption of this gene in the derived strains. The authors also reported a change at the 443rd amino acid in *tlpB*. Although the details of the mutations were different, these genes may be associated with the host change, since they were observed in independent studies, which occurs rarely by chance.

**Table 2:** Mutations reported by previous studies

Gene	TN2-3M	TN2-6M	Reference [19]	Reference [12]
*oppA*	del 1 bp (87451)	del 1 bp (87451)	del 1 bp (1279518 PMSS1)	
*tlpB*	G26W, G275W	del 1 bp (175691)	H443R (PMSS1:SS1)	
*babA*		del 1 bp (989679)		Deletion 6/20 Insertion 4/20 Substitution 3/20

‘del’ stands for a deletion at the genomic position specified within parenthesis. Description under Ref. [12] is the number of samples that harbored the mutation among 20 samples studied

We previously performed a PCR-based study [12] wherein we examined 20 samples of Mongolian gerbils inoculated with *H. pylori*. TM2-6M is one of the strains used in the study. Although the disruption of *babA* by nucleotide deletion/insertion was observed in half of the samples, the deletion/insertion locations and lengths were different. The frequency of disrupted *babA* increased over time after inoculation. This suggested a possible advantage to losing *babA*.

Apart from *babA*, increasing number of nonsense mutations were observed in the current study. The frequencies of the nonsense mutations were 0/6, 2/9, and 3/6 in TN2-1M, TN2-3M, and TN2-6M. Disruption of a gene will not be desirable for the bacteria in its native environment, but it may be selected for if it is advantageous in a new environment. Gene disruption also occurs more easily than gain of a new function by substitution because genes can be broken in various ways, like in *babA*.

### Mutated loci on the protein structure

Protein structure data were available for *ureI* (3UX4) [39] and *virB11* (1NLZ) [40]. We downloaded the data and marked the mutated loci on the structure.

**Fig. 2:**
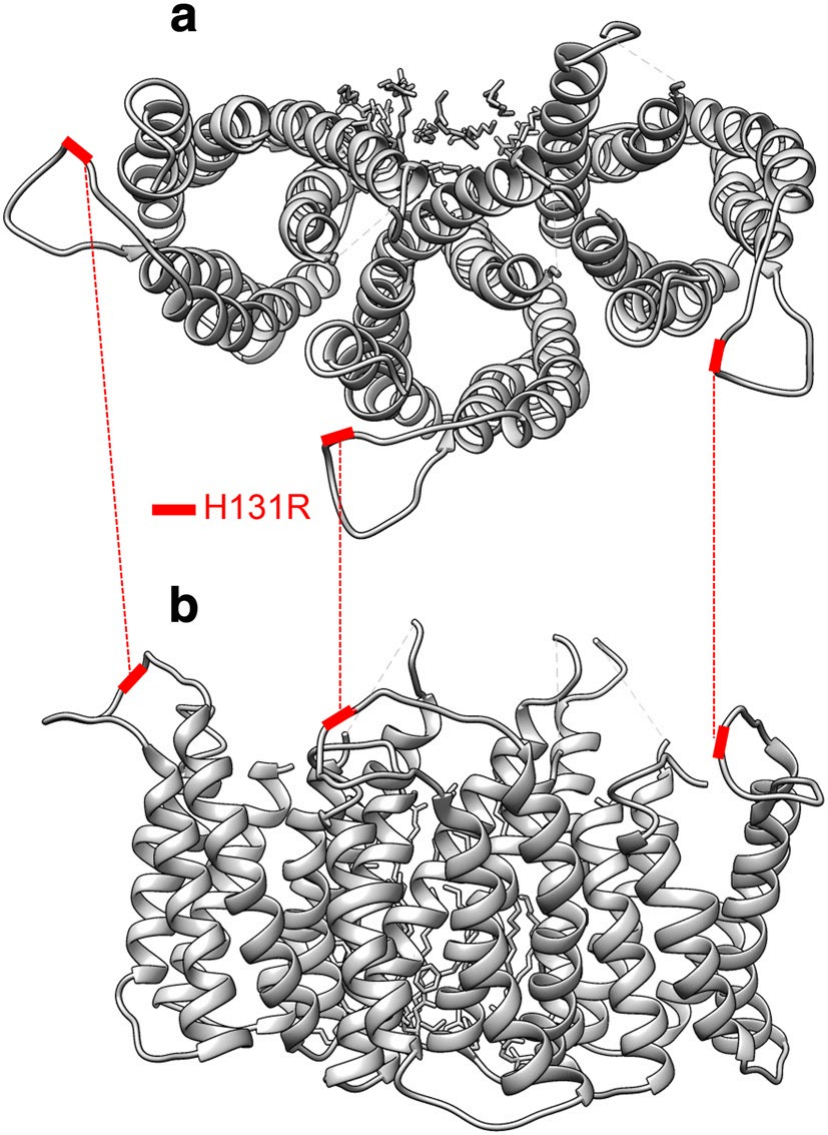
Three dimensional structure of UreI and the location of H131R. The top view (**a**) and side view (**b**) of half of the hexametric ring of UreI channel

**Fig. 3:**
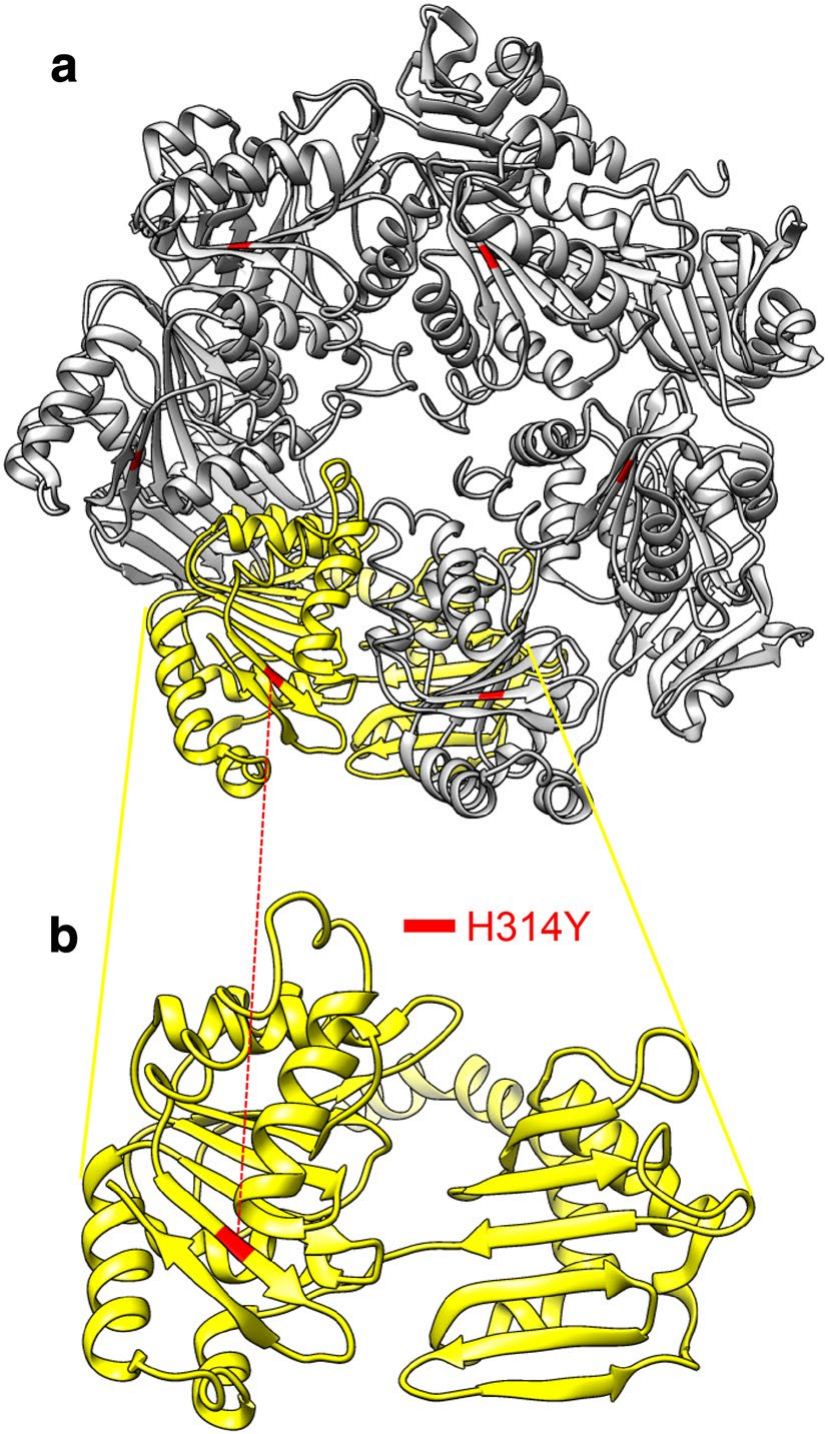
Three dimensional structure of VirB11 and the location of H314TR. The top view of the hexametric assembly (**a**) and magnification of one protomer (**b**)

UreI channel consists of six protomers that form a hexametric ring. Figure 2 shows the half of the hexametric ring and the location of H131R in each protomer. H131 is located in periplasmic loop 2 (PL2). Previous study substituted amino acids of various loci in PL2 and reported that H131R hampered urea transportation in *Xenopus laevis* oocytes [41]. Figure 3 shows the location of H314Y in VirB11. VirB11 also form a hexametric assembly. H314Y is located in a b-sheet near the end of the protomer, however, no function is reported about this locus.

Structure data of TlpB was also available but G26W and G275W were outside of the analyzed region. According to protein domain information, G26W is contained in the tm1 (transmembrane helices 1) and G275W is in HAMP (histidine kinase, adenylyl cyclase, methyl-binding protein, phosphatase) domain. Tm1 mediates signal transmission across the membrane by piston-like motion of tm2 relative to tm1. HAMP domain is supposed to constitutes a switch region that translates the piston-like motion into a different type of transition within the distal portions [42]. Therefore, mutations G26W and G275W may influence on the function of the chemoreceptor for acidity and urea.

## Conclusions

We compared *H. pylori* genomes between original TN2wt and three strains retrieved after inoculation to Mongolian gerbils. We identified mutations in 21 loci of 17 genes of the post-inoculation strains. Mutated genes included *babA*, *tlpB*, and *gltS*, which is known to be associated with adaptation to murine. Other mutations were involved with chemoreceptor, pH regulator, and outer membrane proteins, which also have potential to influence on the adaptation to the new host.

## Data Availability

Genome sequence data of TN2wt is available from GenBank under the Accession number AP019730.
